# COMMUNITY ORGANIZATIONS’ OUTREACH AND SERVICE DELIVERY TO OLDER ADULTS LIVING WITH DEMENTIA AND FAMILY CAREGIVERS

**DOI:** 10.1093/geroni/igae098.2860

**Published:** 2024-12-31

**Authors:** Kathy Lee, Jessica Cassidy, Soeun Jang

**Affiliations:** The University of Texas Arlington, Arlington, Texas, United States; The University of Texas Arlington, Arlington, Texas, United States; University of Texas at Arlington, Arlington, Texas, United States

## Abstract

The current study examined various types of social services for older adults living with Alzheimer’s disease or related dementia (ADRD) and their family caregivers, as well as the methods of delivery employed by home- and community-based organizations. Using a qualitative study design, this study aimed to identify strategies for enhancing support for older adults with ADRD and their family caregivers. A convenience sample method was employed to recruit service providers from nine home- and community-based organizations working closely with older adults living with ADRD and their family caregivers. Semi-structured, individual interviews were conducted online and transcribed verbatim by professionals. Three themes emerged: (1) culturally tailored outreach and services, (2) engagement of older adults living with ADRD and their family caregivers, and (3) collaboration efforts between home- and community-based organizations. Our findings revealed that organizations have tried creative ways to reach their population and educate people on dementia and dementia care. Especially, nonconventional methods (e.g., religion-based outreach or edutainment – an approach combining educational content with entertaining/easy-to-understand elements, such as storytelling or drama) were more frequently discussed among organizations serving people from diverse cultural and ethnic backgrounds. While many organizations have adopted technology to deliver services, many mentioned technologies were not a long-term solution, specifically for older adults with ADRD, as non-verbal cues such as eye contact and physical touch were considered critical for communication and providing care for older adults with ADRD.

